# Screening Coverage Needed to Reduce Mortality from Prostate Cancer: A Living Systematic Review

**DOI:** 10.1371/journal.pone.0153417

**Published:** 2016-04-12

**Authors:** Ahmad K. Rahal, Robert G. Badgett, Richard M. Hoffman

**Affiliations:** 1 Department of Internal Medicine, Kansas University School of Medicine, Wichita, Kansas, United States of America; 2 Department of Internal Medicine, Division of General Internal Medicine, University of Iowa Carver College of Medicine, Iowa City, Iowa, United States of America; Hvidovre Hospital, DENMARK

## Abstract

**Introduction:**

Screening for prostate cancer remains controversial because of conflicting results from the two major trials: The Prostate, Lung, Colorectal and Ovarian Cancer (PLCO) screening trial and the European Randomized Study of Screening for Prostate Cancer (ERSPC).

**Objective:**

Meta-analyze and meta-regress the available PSA screening trials.

**Methods:**

We performed a living systematic review and meta-regression of the reduction in prostate cancer mortality as a function of the duration of screening provided in each trial. We searched PubMed, Web of Science, the Cochrane Registry, and references lists from previous meta-analyses to identify randomized trials of PSA screening. We followed PRISMA guidelines and qualified strength of evidence with a GRADE Profile.

**Results:**

We found 6 trials, but excluded one that also screened with trans-rectal ultrasound. We considered each ERSPC center as a separate trial. When pooling together all 11 trials we found no significant benefit from screening; however, the heterogeneity was 28.2% (95% CI: 0% to 65%). Heterogeneity was explained by variations in the duration of serial screening (I^2^ 0%; 95% CI: 0% to 52%). When we analyzed the subgroup of trials that added more than 3 years of screening (range 3.2 to 3.8) we found a significant benefit for screening with risk ratio 0.78 (95% CI 0.65–0.94; I^2^ = 0%; 95% CI: 0% to 69%) and a number needed to invite for screening of 1000. We downgraded the quality of evidence to moderate due to our retrospective identification of subgroups and limited data on control group screening.

**Conclusions:**

Adequate duration of screening reduces mortality from prostate cancer. The benefit, while small, compares favorably with screening for other cancers. Our projections are limited by the moderate quality of evidence.

## Introduction

Screening for prostate cancer remains controversial due to conflicting results from the European Randomized Study of Screening for Prostate Cancer (ERSPC) and the Prostate, Lung, Colorectal and Ovarian Cancer Screening Trial (PLCO) [1–2]. The Cochrane Collaboration reviewed the five relevant trials and found no reduction in death from prostate cancer; however, the heterogeneity was moderate (I^2^ = 46%) [3]. Accordingly, practice guidelines conflict [4–7]. Resolution of these conflicts is needed to inform public opinion. Our objective was to determine the reduction in mortality from prostate cancer by serial measurement of the prostate-specific antigen (PSA) rather than usual care among men aged 50 to 74 years. In this meta-analysis, we treated each ERSPC center as a separate trial and explored heterogeneity of results with meta-regression of the total duration of screening.

We used many of the methods recently proposed by Elliott et al [8] in their call for living systematic reviews. Our use of openMetaAnalysis supports Elliott’s vision of data sharing and crowd-participation [9–10].

## Methods

### Living Systematic Review

We conducted a living systematic review using openMetaAnalysis [9–10]. Living systematic reviews has been proposed as a solution to the problems of tradition, static meta-analyses becoming outdated. OpenMetaAnalysis is an open-source, cloud-based approach that implements a living systematic review based on the concepts of the Cochrane, PRISMA (Preferred Reporting Items for Systematic Reviews and Meta-Analyses) [11] and GRADE (Grading of Recommendations Assessment, Development and Evaluation) [12]. In a living systematic reviews author’s work is shifted from searching to interpretation by building on the studies included in prior reviews. It also commoditizes methods by building on prior reviews so clinicians can participate in the interpretation using a quickly updated methodology (for example, using the Hartung-Knapp estimator and confidence intervals for heterogeneity). We encourage colleagues to use our data online and add to this review as new studies or perspectives emerge [13].

### Eligibility Criteria for Trials

We included randomized control trials of PSA screening for prostate cancer that reported mortality due to prostate cancer. We excluded trials with multiple methods of screening if we could not isolate the effect of screening using PSA measurements.

### Information Sources

We reviewed all trials included in the last 3 meta-analyses of PSA screening [3–5]. We also used PubMed, Cochrane Central Register of Controlled Trials, and Web of Science.

### Search Strategy

We searched PubMed, the Cochrane Central Register of Controlled Trials, and Web of Science through March 2016 for newer trials that were published since the search date of the meta-analysis by the Canadian Task Force (August, 2014) [5]. PubMed search terms used were prostate cancer, mortality and prostate-specific antigen for all fields. We also searched Web of Science for citations that cited the seminal trials by the PLCO or the ERSPC and had the text string ‘random’ in the title or abstract. Lastly, we employed automated alerts that signal candidate trials or reviews released on PubMed or ClinicalTrials.gov.

### Data Abstraction

Due to the heterogeneity of study designs and screening implementation, we treated each study site in the ERSPC as a separate trial. We had to confine our analysis of the ERSPC trial to results at 11 years of follow-up and males in the core age group of 55 to 69 years of age as only these results were reported separately for each center [2]. For the PLCO, we used the results after 9 years of follow-up [1]. We obtained information about trial design, adherence to and contamination of screening from the original [1–2] as well as subsequent [14–16] publications on these trials. Data was abstracted into spreadsheets online [13].

**Fig 1:**
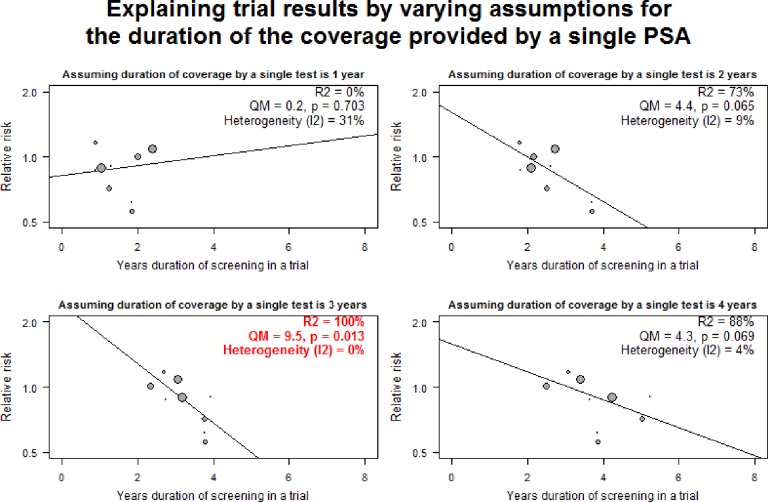
Comparison of ability to explain trial results by varying assumptions for the duration of the coverage provided by a single PSA.

The total duration of screening (“Duration-Total”) for each trial was the estimated years of coverage by serial measurement of PSA levels. To determine the total duration of a screening program, we first estimated the years of duration of coverage provided by a single PSA test (“Duration-Single”). To determine the Duration-Single, we compared the ability of assuming Duration-Single was 1, 2, 3, or 4 years to explain the variations in the results of the trials (Fig 1). For example, when we assumed a duration of 1 year, there was no correlation between the duration of screening and the results of the trials. In addition, moderate heterogeneity of results of the trials remained. However, assuming Duration-Single was 3 years led to the least residual heterogeneity of the results of the trials (I^2^ 0%; 95% CI: 0% to 52%) and the highest value for the Q_M_ test for significance of association. Duration-Total was calculated from Duration-Single after being adjusted for compliance and contamination in the screened and control subjects, respectively, in the trials. Details of the calculation of Duration-Total and Duration-Single are in S1 File.

In addition to abstracting trials, we abstracted prior systematic reviews to identify PSA screening for prostate cancer [3,5,17]. We constructed two tables: first, a table of trials that reconciled the trials included by each review, and second, a table of conclusions of prior trials that reconciled the results and their heterogeneity.

### Statistical Analysis

We conducted meta-analysis with the Dersimonian-Laird inverse variance estimator using the Hartung-Knapp adjustment for calculating relative risk [18]. For meta-regression, we measured the association between the duration of a screening program and the reduction in mortality from prostate cancer. We performed a pre-specified meta-regression of the natural log of the relative risk of mortality by the calculated years of coverage added by screening. We determined the threshold number of years where both the point estimate and the upper end of its 95% confidence interval for the relative risk of mortality due to prostate cancer were less than 1. All analyses were done with R [19].

### Assessment of Quality

We formally assessed trials with the Cochrane Risk of Bias Tool [20]. We qualified conclusions with the Grade Working Group’s Evidence Profile [21]. We created a Summary of Findings Table [21]. We tabulated our conclusions in comparison to those of prior meta-analyses as recommended by Riaz [22]. We used the results from the Summary of Findings table to create a patient information page in the format of a “Drug Facts Box” proposed by Schwartz et al [23].

## Results

**Fig 2:**
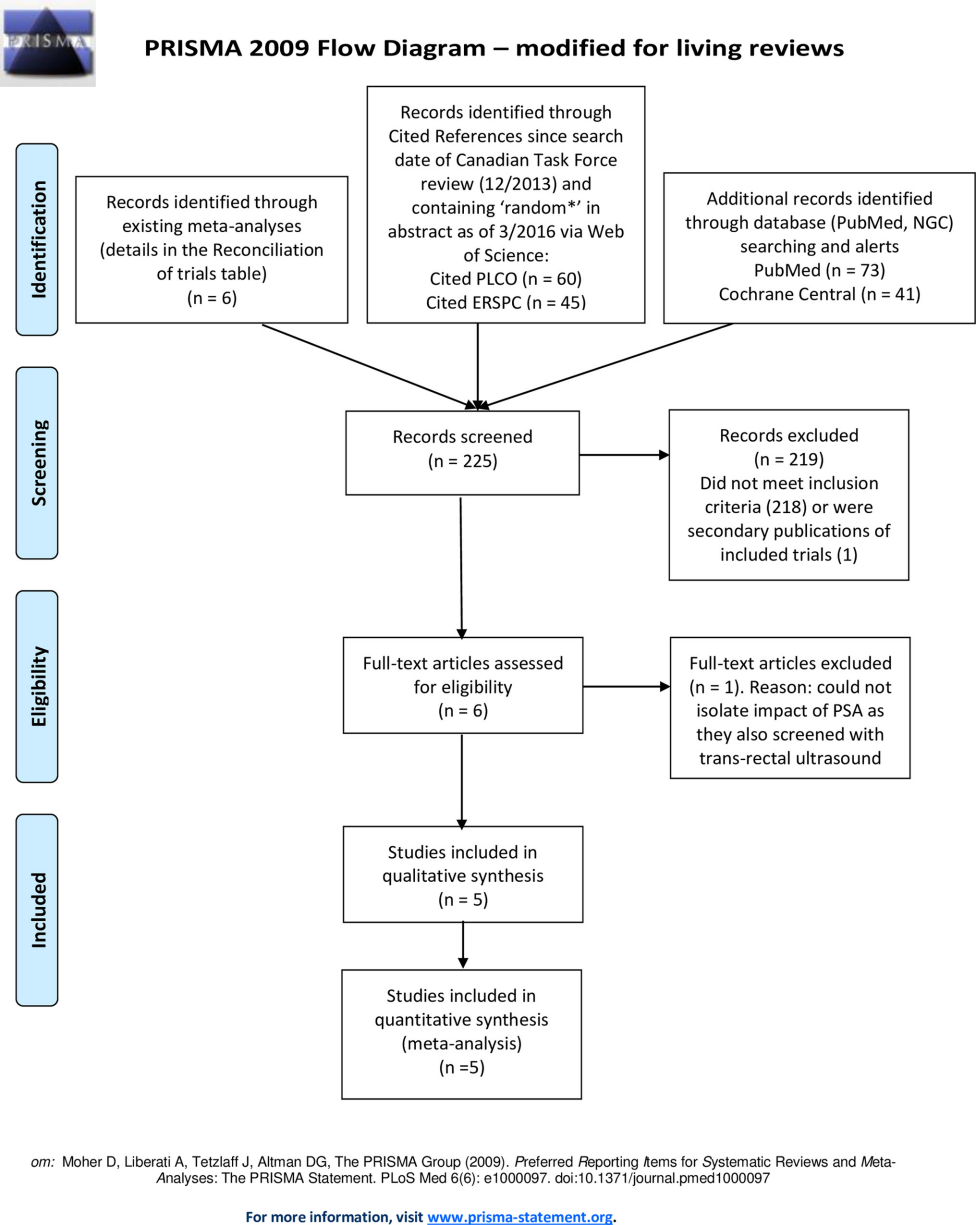
Flow diagram for included studies of this meta-analysis.

We identified a total of 225 citations, including 6 from reference lists of meta-analyses, 73 from the PubMed search, 41 from Cochrane Central, and 105 from Web of Science. We excluded 219 by screening titles and abstracts. Of those 218 did not meet inclusion criteria and 1 was a secondary publication of an included trial. After reviewing the full text of the remaining 6 studies, 1 study was excluded [24] because screening included trans-rectal ultrasound along with PSA. We included 5 randomized controlled trials of screening for prostate cancer by the PSA in our final quantitative analysis [1–2, 25–27], details are in the PRISMA flow diagram online (Fig 2). A Table of Reconciliation of Trials included in our review compared with prior meta-analyses is available online [13]. The descriptions of the trials are in the PICO (patient problem or population (P), intervention (I), comparison (C), outcome (s) (O))and Risk of bias tables is available online as well [13].

**Fig 3:**
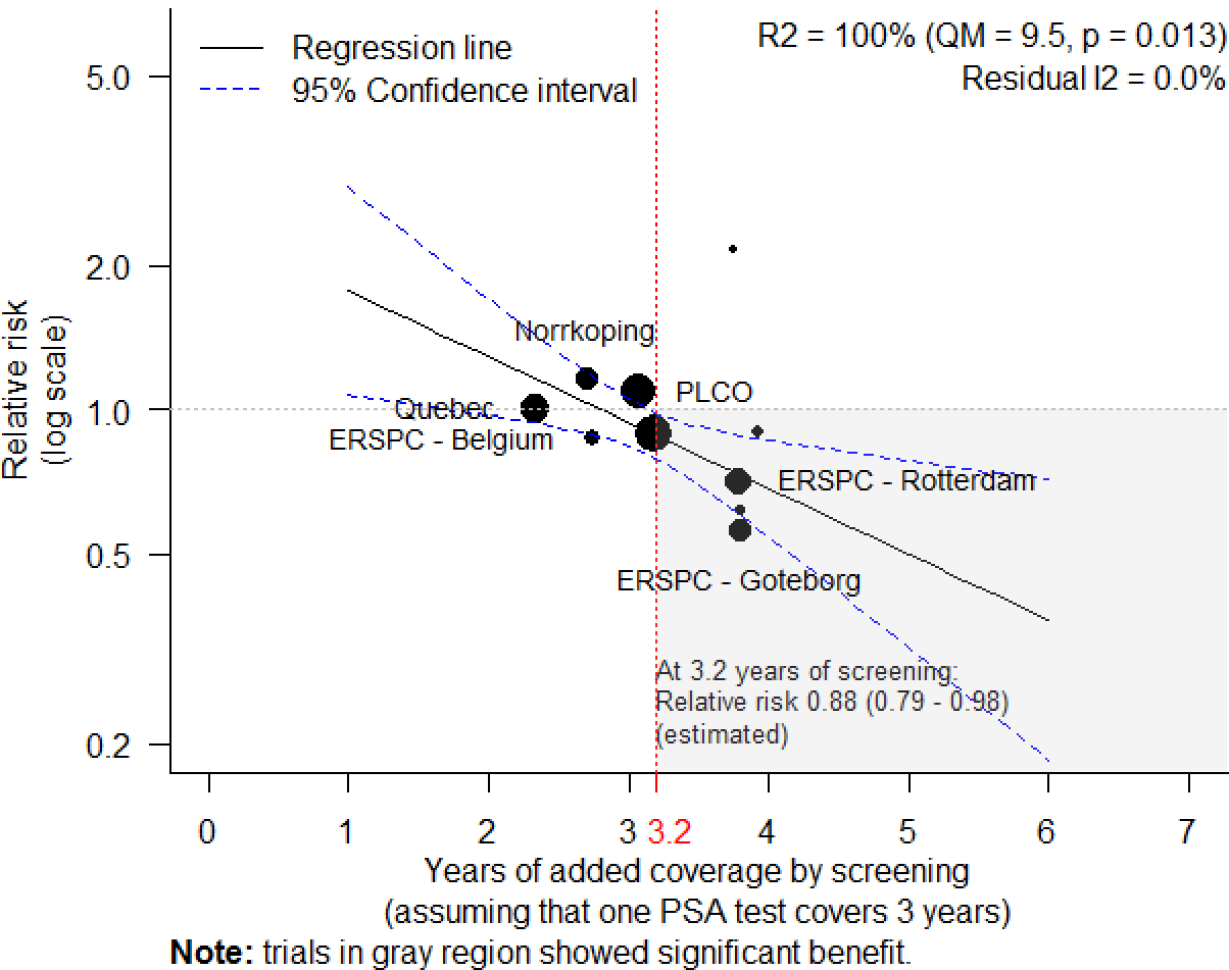
Determining the minimum duration of a screening program required to reduce death from prostate cancer.

When all trials are pooled together there is no significant benefit from screening (Table 1). Heterogeneity of the point estimates of the results “might not be important” according to the scale used by the Cochrane Collaboration [28]; however, the results of the largest trials conflict. As noted in the methods, we assumed that the duration of coverage of a single PSA was 3 years as this value best explained the variation in results among the trials (Fig 1). With the assumption of 3 years, I^2^ was 0% (95% CI: 0% to 52%). We then determined the threshold in years for the total duration of coverage by a screening program (Fig 3). As shown in Fig 3, reduction in mortality due to prostate cancer is expected to occur when more than three years of screening is provided. For sensitivity analysis, when duration of coverage is assumed to be 2 or 4 years, the threshold for years of screening becomes 2.6 and 3.9 years, respectively.

**Table 1:** Summary of meta-analyses for overall benefit.

	Mortality due to prostate cancer-Relative Risk (95% CI)	Heterogeneity I^2^ (95% CI)
**Current meta-analysis (ERSPC limited to core age group)**	**0.89 (0.76 to 1.04)**	**28% (0% to 65%)**
**Cochrane meta-analysis**		
**As reported**	**1.00 (0.86 to 1.17)**	**46% (CI not reported)**
Reanalyzed*	**1.00 (0.83 to 1.21)**	**46% (0% to 80%)**

*Reanalyzed with Hartung-Knapp adjustment to the Dersimonian-Laird inverse variance

**Fig 4:**
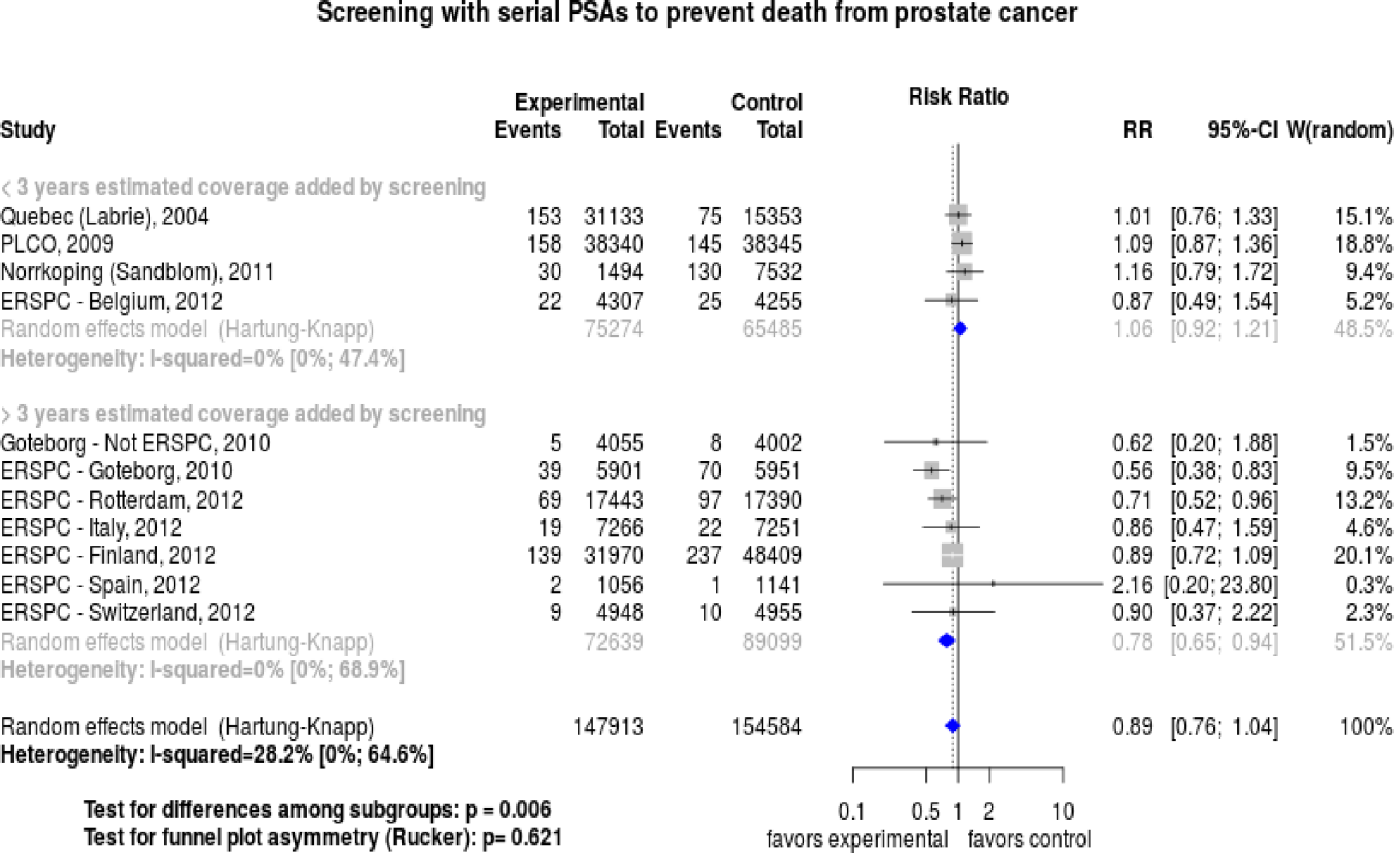
Forest plot of trials sub-grouped by estimated duration of monitoring.

Based on the exploration of heterogeneity, we divided the trials into those that added more than 3 years of monitoring (range 3.2 to 3.8) versus those that added less (range 2.1 to 2.7). These subgroups are shown in the forest plot (Fig 4). The subgroup analysis found significant reduction of the risk ratio with 95% confidence intervals (CIs) of 0.78 (0.65–0.94) among the trials a long duration of screening and there was no heterogeneity of results in the subgroups (Fig 4). The number need to invite (NNI) to screen among the subgroup of trials adding more than 3 years of monitoring was 1000 and the NNI was 194 in the older cohort within the Göteborg trial [27].

The risk of bias assessment for each trial are online in the interactive risk of bias table [13]. We deemed the methodology of trials to be of unclear risk of bias due to high or unclear attrition of subjects in all trials.

**Fig 5:**
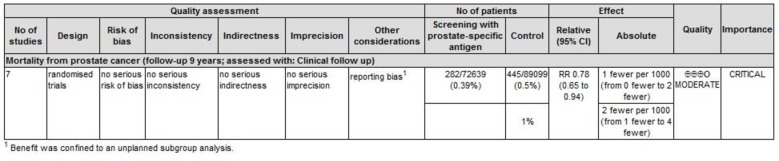
GRADE evidence profile.

We deemed the overall quality of evidence as moderate (Fig 5). This was largely due to our retrospective assembling of subgroups.

Lastly, we deemed the methodology of trials to be of unclear risk of bias due to high or unclear attrition of subjects in all trials. Details of the quality of evidence are available in the risk of bias and summary of findings tables online [13]. We created a patient information handout based on these results. The handout is online and licensed with the GNU Version 3 General Public License for free reprinting [13].

## Discussion

We found significant benefit from screening among trials with sufficiently long duration of PSA screening compared to control groups. 'Adequate' was adding more than 3 years duration of screening. There was no heterogeneity of results in this subgroup. We deemed the quality of evidence as moderate.

There are implications of these projections. First, this result suggests that benefit is gained without requiring annual screening, which is consistent with studies that have modeled data from non-randomized cohorts of men and suggested benefit is affected by the interscreening interval [29–32]. Less frequent screening is recommended for High Value care by the American College of Physicians [33]. While we project benefit from interscreening intervals up to four years, we do not address the optimal interscreening interval. Wu et al addressed this with a decision analysis based on the ERSPC results [32]. While Wu projected benefits from inter-screen intervals as long as 8 years, the strongest benefit was an interval of one year. Second, the NNI compares with screening for other cancers by being both more [34] and less [35] favorable.

On first glance, our results seem to surprisingly imply that a single PSA test, as compared to no testing, will reduce death from prostate cancer. However, no trial studied this scenario as in all models we tested, all trials that found benefit were estimated to add more years of monitoring than added by a single test. The decision analysis by Wu projected benefit with a single PSA measurement at 65 years of age; however, number needed to screen (NNS) was very high at 2500 men. The NNS fell to 536 if a second PSA was done. The value of a single screen is being prospectively tested in the large Cluster randomized trial of PSA testing for Prostate cancer (CAP) [36].

Our results are limited by several factors. First, our explorations of subgroups was retrospective. Second, we estimated the mean number of non-protocol PSA tests among the non-screened men as this number was only reported in the PLCO trial. Third, we cannot confidently quantify the benefit that is projected from more than 4 years of testing. This is due to standard meta-regression presuming a linear relationship between the years of coverage and the natural log of the relative risk. However, benefit might diminish with prolonged testing. Fourth, other factors such as degree of pre-trial screening likely affect the results of trials; however, these factors were not sufficiently reported for us to analyze. Fifth, we cannot measure reduction in all-cause mortality as this has not been reported for individual ERSPC centers. Additionally, the trials included few African-American males. In this group, the incidence of death from prostate cancer is doubled which potentially reduces the NNS from 1000 to approximately 500 for African-Americans [37]. Lastly, we did not account for the quality of life after treatments for prostate cancer which may diminish perceived benefit [38].

Living systematic review have been proposed to improve the dissemination of medical evidence [8, 39]. OpenMetaAnalysis is one method and supports collaborative, team science by facilitating communication between contributions, documentation, and version control for a single or alternative versions. In addition, the revised search strategy of a living systematic review allows effort to be shifted from searching to interpretation. In short, living systematic reviews commoditize the maintenance and updating of reviews so that savvy clinical faculty and advanced trainees can contribute.

As compared to prior meta-analyses, our review explain contradictory results among trials by considering the duration of screening implemented in each trial and in each center of the ERSPC. A comparison to prior meta-analyses is in the Reconciliation of Conclusions which is online and replicated in Table 2 [19].

**Table 2:** Reconciliation of conclusions with prior meta-analyses.

	Current living systematic review	USPSTF (Chou), 2014 [17]	CTFPHC, 2014 [5]	Cochrane (Ilic), 2013 [3]
**Conclusions**	**Benefit >3 years duration of screening**	**No benefit**	**No benefit, trials not pooled**	**No benefit in pooled data**
**Heterogeneity**†	**None (I**^**2**^ **= 0%)**	**Not reported**	**Not applicable**	**Moderate (I**^**2**^ **= 46%)**

Concept for table by Irbaz B. Riaz, MD. Arizona Health Sciences Center [22]

† Heterogeneity descriptions are by the Cochrane Collaboration [28]

## Conclusion

In summary, our analyses suggest a small reduction in mortality from prostate cancer due to screening. The trials are homogenous in results after controlling for study level factors. The dependence of the conclusions on estimates of non-protocol screening reinforces the need for better reporting of processes in screening studies. We encourage colleagues to use and augment this living systematic review.

## Supporting Information

S1 File Appendix. Details of the calculation of Duration-Total and Duration-Single.
(PDF)

S2 File The PRISMA 2009 Checklist. (PDF)
