# Supplementary material for: Screening Coverage Needed to Reduce Mortality from Prostate Cancer: A Living Systematic Review
Source: PLoS One. 2016 Apr 12;11(4):e0153417. doi: 10.1371/journal.pone.0153417 (PMC4829241; doi:10.1371/journal.pone.0153417)
Supplement: S1 File — Details of the calculation of Duration-Total and Duration-Single. (PDF) [file pone.0153417.s001.pdf]

Appendix

Details of the calculation of Duration-Total and Duration-Single

We created a measure for the effective increase in testing with prostate specific antigen (PSA) due to screening as a function of the years of coverage provided by one test ("DURATION-SINGLE"), compliance in the screening group, and contamination in the control group. The definitions used for these values were:

DURATION-SINGLE is the projected duration in years of coverage provided by a single PSA test

MTS was the mean number of tests per male in the screened group

MTC was the mean number of non-protocol tests per male in the control group. This was calculated as the MTS times the reported rate of contamination among control subjects. This calculation was based on the reported MTS, contamination rate, and MTC for the PLCO of 5, 52%, and 2.7, respectively.

With these values, we used the following equations:

DURATION-SINGLE-EFFECTIVE is the minimum of DURATION-SINGLE and PSA testing frequency in the trial. For example, if the interscreening interval is shorter than the projected duration of coverage provided by a single PSA, then the DURATION-SINGLE-EFFECTIVE is the same is the interscreening interval

DURATION-TAIL is the additional coverage by the last PSA done in the trial. It is calculated as (DURATION-SINGLE-EFFECTIVE - frequency of PSA testing).

DURATION-TOTAL is the total number of years of coverage in a trial. This is calculated as DURATION-SINGLE-EFFECTIVE times the number of tests performed in a trial plus COVER_TAIL

The following, final equation calculated the total duration. Note that both DURATION-SINGLE-EFFECTIVE and DURATION-TAIL were both adjusted by the compliance and contamination in the screening and control groups, respectively.

DURATION_TOTAL = DURATION-SINGLE-EFFECTIVE * (MTS - MTC) + DURATION_TAIL * (Compliance - contamination)

Sample calculations are online at http://openmetaanalysis.github.io/Prostate-cancer-screening-with-prostate-specific-antigen/
